# The 5S-5M-5C schematic: transforming primary care inputs to outcomes in low-income and middle-income countries

**DOI:** 10.1136/bmjgh-2018-001020

**Published:** 2018-10-02

**Authors:** Asaf Bitton, Jeremy H Veillard, Lopa Basu, Hannah L Ratcliffe, Dan Schwarz, Lisa R Hirschhorn

**Affiliations:** 1 Ariadne Labs, Brigham & Women’s Hospital and Harvard T.H. Chan School of Public Health, Boston, Massachusetts, USA; 2 Health, Nutrition and Population Global Practice, World Bank Group, Washington, District of Columbia, USA; 3 Department of Medicine, Johns Hopkins School of Medicine, Baltimore, Maryland, USA; 4 Northwestern University Feinberg School of Medicine, Chicago, Illinois, USA

**Keywords:** primary care, primary health care, universal health coverage, LMIC, quality

**Summary box**

- High-quality primary healthcare (PHC) is critical to achieving universal health coverage (UHC).
- Primary care (PC) clinical services make up an important part of the value that a PHC approach offers.
- There is a dearth of understanding about how to transform system inputs into the desired PHC outcomes.
- We propose a schematic detailing the mechanisms and system-level functions required to transform inputs into better outcomes in PC clinical systems.
- This schematic has important implications for the global research and policy agendas needed to achieve UHC by 2030. Particular emphasis should be placed on better measurement of the mechanisms described here, stronger data systems to translate measurement into improvement and private sector engagement and innovation to scale improvements in PC service delivery.

## Introduction

High-quality primary healthcare (PHC) is the most effective way to deliver person-centred, promotive, preventive and curative services to meet the majority of a population’s health needs.^1^ PHC is critical to improving population health, making health systems more equitable and resilient and promoting global health security. Furthermore, PHC is instrumental to achieving quality universal health coverage (UHC) and meeting the Sustainable Development Goals. However, as the global community marks the 40th anniversary of the Alma Ata Declaration in 2018,^2^ a significant gap remains between the original Declaration’s aspirational vision and the current reality of PHC throughout the world. PHC remains a neglected area of investment in most low-income and middle-income countries (LMICs), with limited prioritisation in public sector spending, poor integration with other sectors and alarming deficiencies in the quality of primary care (PC) clinical services delivered.^3^ Visits in PC are short, diagnoses frequently incorrect and treatments often unnecessary or harmful.^4^ Community priorities around healthcare needs are often not elicited within PHC and feedback from patients and communities is rarely sought.

To support PHC strengthening and improvement globally, the Primary Health Care Performance Initiative (PHCPI) was launched in 2015.^3^ This initiative works to catalyse PHC improvement in LMICs through better measurement, knowledge dissemination, country engagement and advocacy.^5^ With input from a variety of stakeholders, PHCPI has developed a conceptual framework to guide this work, outlining the key systems, inputs and service delivery components needed to produce better PC-related outputs and outcomes.^5 6^

Central to the PHCPI framework are the four functions of high-quality PC services, described by Barbara Starfield in 1994: first-contact accessibility, continuity, comprehensiveness and coordination or ‘the 4C’s’.^7^ Two decades later, in the midst of the Ebola epidemic, Paul Farmer identified four critical inputs that were lacking in Ebola-affected countries and many other LMICs, and which are also foundational components of the PHCPI framework: systems, space, staff and stuff or ‘the 4S’s’.^8^ Despite decades of investments into these inputs, there has been limited success in converting these 4S’s into equitable, effective PC systems that provide the 4C functions, a shortcoming that has contributed greatly to PC’s unfulfilled potential.

**Figure 1:**
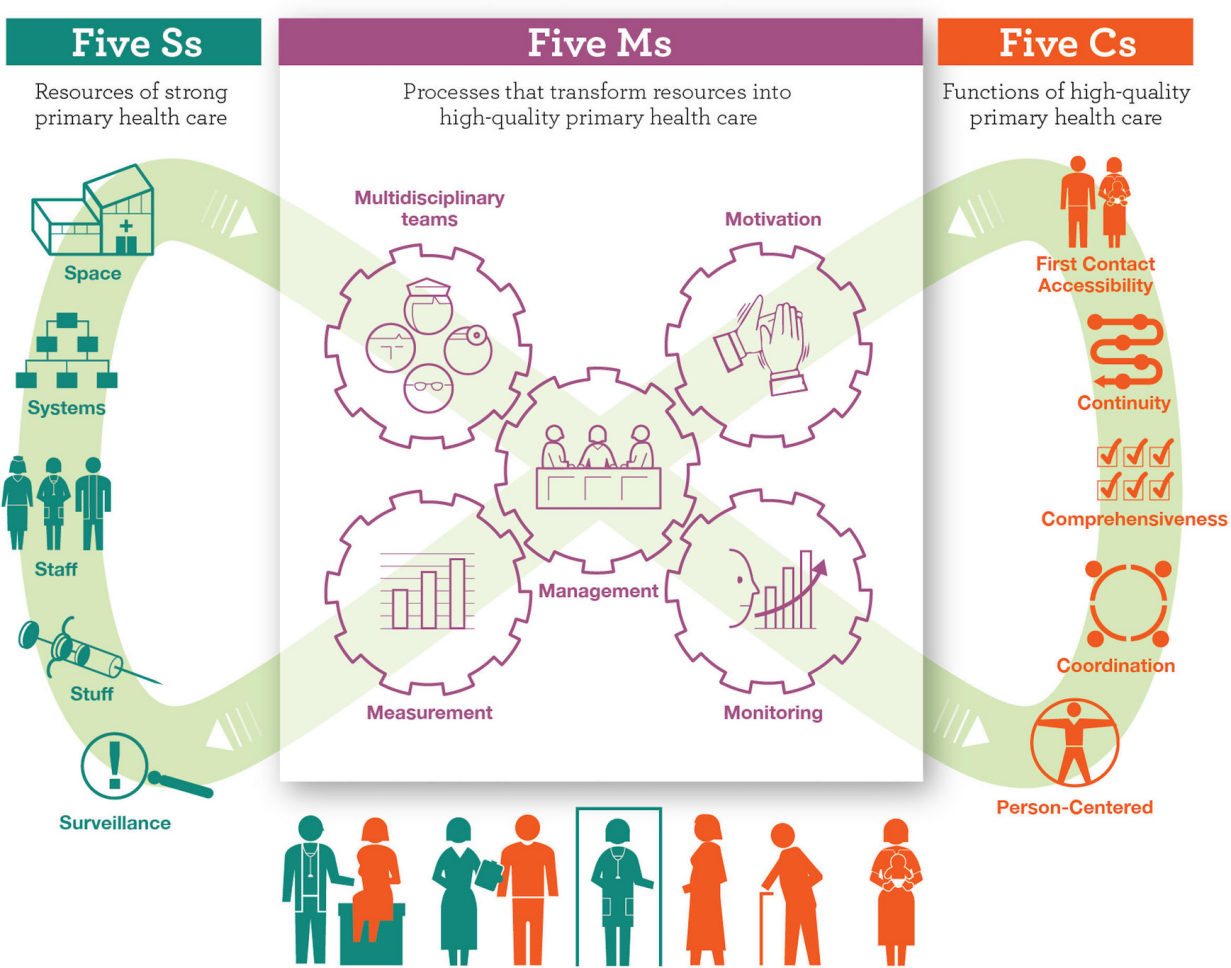
The 5S-5M-5C schematic.

In this paper, we build on this previous work to present a simplified schematic derived from the PHCPI framework—termed ‘5S-5M-5C’ (figure 1)—which describes essential service delivery mechanisms for transforming key inputs into functional and equitable PC systems. Recognising the importance of these often-undervalued mechanisms and better understanding how to measure and improve them, will be critical for making progress towards the goal of quality UHC for all.

## Transforming inputs into better health

### The 5S’s

Traditional health system strengthening efforts have focused on ensuring that healthcare providers are available, equipped with needed medications and supplies, appropriately trained, sufficiently financed, effectively governed and have the information systems required to deliver care—Farmer’s ‘4S’s’.^8^ In addition, the Ebola epidemic highlighted the need for stronger *surveillance* within PC, including the capacity to identify emerging threats and continuously assess and respond to communities’ needs over time. We have incorporated surveillance as a fifth ‘S’ in our schematic.

### The 5C’s

Successful PC systems, by definition, deliver Starfield’s 4C functions. They ensure that services are accessible when and where people need them and in an affordable manner, that continuous, longitudinal patient–provider relationships are fostered and that health information is effectively transferred across time and system levels. They coordinate an individual’s journey through a complex health system, optimising the provision of preventative, chronic management and acute curative services to meet the healthcare needs of families and communities.^7^ Our schematic highlights a fifth ‘C’—that PC must be *person-centred*, meaning that people should be known as a whole person by their regular care provider, feel that their needs and preferences are respected and that their care is effective in meeting expectations and building trust in the PC system.^9^

### The 5M’s

To bridge the gap between the ‘S’s’ and ‘C’s’ and ensure that all people, everywhere receive equitable and high-quality PC services, our schematic defines five critical service delivery mechanisms, termed the 5M’s: *multidisciplinary teams, motivation, measurement, monitoring and facility and population health management*.

In order to meet the range of individual and population health needs demanded of PC, effective *multi-disciplinary teams* must be established, trained and supported. High-quality PC requires not just individual doctors, but rather teams of providers—including nurses, community health workers, mid-level practitioners and other disciplines such as mental health, nutrition and pharmacy—working together with communities to maintain health.^10^

These multi-disciplinary teams need sufficient *motivation* to function effectively.^11^ Motivation levers can be extrinsic, such as payment systems that remunerate teams with adequate and reliable salaries or performance-based incentives linked to improved health outcomes. Staff members also need supportive supervision to produce intrinsic motivation to sustain the provision of competent, empathic, continuously improving and effective care.

Care teams require continuous *measurement* to inform and drive improvement. Current measurement efforts can be made more effective by reducing the plethora of required but often highly disease-specific indicators, strategically including selected novel indicators and leveraging advances in health information systems to strengthen data quality.

But without active and regular *monitoring* to review and use these data, measurement is an exercise in futility. Empowering teams and managers with data and iterative review processes can drive continuous quality improvement, leading to higher quality PC services.^12^

Finally, *facility and population health* *management* systems must be in place to coordinate effective, efficient care at the facility and community levels. Strong PC needs well-trained, experienced managers and effective management systems, without which care delivery will be insufficiently resourced and planned to translate inputs into functions. Population health management, the proactive ascertainment, assessment and improvement of entire populations’ health, is imperative for planning and linking services between communities and facilities. Community health workers linked to empanelment or rostering mechanisms are a critical component of PC systems in this regard, facilitating strategic resource deployment to address specific health needs and ensuring that no one is left behind.^13^

## Insights for primary healthcare development

The 5S-5M-5C schematic and the underlying conceptual framework on which it is based provide insights for developing PC systems capable of equitably meeting the needs of populations, communities and individuals across the globe. We propose that the presence of the 5S inputs and their effective utilisation through the 5M mechanisms will produce the 5C functions, ensuring effective coverage of high-quality PC services and producing better population health. By logically grouping the service delivery mechanisms required to transform inputs into higher PC functions, the ‘5S-5M-5C’ schematic also lays out an agenda for prioritising investments and research in PC systems, both in the public and private sectors. To be effective, the use of the schematic must be locally adapted to reflect relevant social determinants of health as well as the structure and goals of the national health system. Box 1 provides a country example of successful implementation of key areas in this schematic.

Box 1 **Effective 5S-5M-5C implementation in Costa Rica^16^**

In Costa Rica, healthcare is a human and legal right enshrined in the constitution. Over the past 30 years, a robust public primary care (PC) system has been built and financed to meet the changing health needs of the population. Each person has financial and geographic access to public PC services in their community. Members of community-based PC teams known as *EBAIS* take care of a defined, empaneled population of approximately 4500 people. The EBAIS teams send a community health worker to every household in a given catchment area at least once a year to collect basic information on demographics, health risks and opportunities for health promotion. People deemed at risk of chronic diseases, or who have gaps in care, are encouraged to seek facility-based treatment at their local EBAIS PC facilities staffed by doctors, nurses and pharmacists. Health and quality data are collected using standardised forms and shared with local health area managers, who aggregate the data for national planners. EBAIS staff are regularly measured on the performance outcomes for their catchment area and the results are fed back to them for improvement. Health areas with major equity gaps in performance are prioritised for further investment with more EBAIS teams or other resources in order to narrow those gaps. The system aims to provide not just basic preventive services and promotive education at a community level, but also more comprehensive, accessible care for the chronic non-communicable diseases that make up the largest proportion of disease burden in Costa Rica. The EBAIS coordinate and track referrals to hospital and specialty care. The EBAIS also link up with other social service sectors to promote a comprehensive approach to health within and around the formal health system.

Historically, efforts to strengthen health system performance have overly focused on input-driven initiatives that fund the 4S’s, often through disease-specific vertical programmes. However, adopting a business-as-usual approach will be insufficient to achieve the global goal of UHC in a sustainable, efficient and equitable manner. The path towards UHC is dependent on strong, high-quality PC to support overall PHC goals. In turn, the path towards improving PC systems involves first acknowledging the centrality of the 5M mechanisms for effective service delivery. A research and policy agenda focused on better measuring and strengthening the 5M mechanisms is necessary to discern new ways to drive improvement in PC service delivery, coupled with meaningful support to LMICs to build evidence-informed systems. To address these gaps, PHCPI has launched a research consortium dedicated to developing actionable knowledge and policy recommendations to achieve these goals.^3^

In addition to further clarifying how and what to measure to strengthen PHC, further investments in stronger data and performance monitoring systems are needed as well as analytical tools to use this information to drive improvement. This requires more robust health information systems capable of aggregating data from multiple sources, including both public and private care delivery systems and providing timely and actionable data to decision makers and health workers to modify services at the local level. Initiatives such as the Health Data Collaborative^14^ have started this important work, but there is still much progress to be made.

Finally, while much of PC in LMICs is provided by the public sector, there is a large role for private sector engagement and innovation, both as direct service providers and through public–private partnerships.^15^ There are many insights to be taken from private sector systems’ design and management, particularly related to the 5M mechanisms, that can improve efficiencies and scale strategies, while also ensuring that equity is prioritised in both public and private sector service delivery. Without incorporating these lessons and innovations and directly including private sector partners in the delivery of PC, the goal of UHC will likely be unachievable.

## Conclusion

The 5S-5M-5C schematic derived from the PHCPI framework provides a simplified, structured approach to understanding how investments in essential inputs (5S’s) must be accompanied by investments in the key service delivery mechanisms (5M’s), to enable the equitable delivery of person-centred, high-quality PC (5C’s) to populations, communities and individuals. As the world celebrates 40 years since the Alma Ata Declaration and embarks on the path towards quality UHC by 2030,^10^ we believe that this schematic can help guide the policy and implementation conversations necessary to achieve this important global goal.

## References

[1] Macinko J, Starfield B, Erinosho T The impact of primary healthcare on population health in low- and middle-income countries. J Ambul Care Manage 2009;32:150–71. 10.1097/JAC.0b013e3181994221 19305227

[2] Beard TC, Redmond S, Alma-Ata Dof Declaration of Alma-Ata. Lancet 1979;313:217–8. 10.1016/S0140-6736(79)90622-6

[3] Primary health care performance initiative. www.phcperformanceinitiative.org

[4] Das J, Hammer J Quality of primary care in low-income countries: facts and economics. Annu Rev Econom 2014;6:525–53. 10.1146/annurev-economics-080213-041350

[5] Veillard J, Cowling K, Bitton A, et al Better measurement for performance improvement in low- and middle-income countries: the primary health care performance initiative (phcpi) experience of conceptual framework development and indicator selection. Milbank Q 2017;95:836–83. 10.1111/1468-0009.12301 29226448 PMC5723717

[6] Bitton A, Ratcliffe HL, Veillard JH Primary health care as a foundation for strengthening health systems in low- and middle-income countries. J Gen Intern Med 2017;32:566–71. 10.1007/s11606-016-3898-5 27943038 PMC5400754

[7] Starfield B Is primary care essential? Lancet 1994;344:1129–33. 10.1016/S0140-6736(94)90634-3 7934497

[8] Farmer P Diary: Ebola. London Rev Books 2014;36:38–9.

[9] World Health Organization. Framework on integrated, people-centred health services 2016.

[10] WHO. Global strategy on human resources for health: Workforce 2030: 2010. 10.1186/s12960-024-00940-x PMC11450981 39363378

[11] Dugani S, Afari H, Hirschhorn LR, et al Prevalence and factors associated with burnout among frontline primary health care providers in low- and middle-income countries: a systematic review. Gates Open Res 2018;2:4 doi:10.12688/gatesopenres.12779.3 29984356 10.12688/gatesopenres.12779.3 PMC6030396

[12] World Health Organization. Monitoring the building blocks of health systems: a handbook of indicators and their measurement strategies. Geneva: World Health Organization, 2010.

[13] Ballard M, Schwarz R Employing practitioner expertise in optimizing community healthcare systems. Healthcare 2018. doi: 10.1016/j.hjdsi.2018.08.003. [Epub ahead of print: 23 Aug 2018]. 10.1016/j.hjdsi.2018.08.003 30146473

[14] Health data collaborative. https://www.healthdatacollaborative.org/

[15] Wadge H, Roy R, Sripathy A, et al How to harness the private sector for universal health coverage. Lancet 2017;390:e19–e20. 10.1016/S0140-6736(17)31718-X 28651883

[16] Pesec M, Ratcliffe HL, Karlage A, et al Primary health care that works: the Costa Rican Experience. Health Aff 2017;36:531–8. 10.1377/hlthaff.2016.1319 28264956

